# Supplementary material for: The role of competition versus cooperation in microbial community coalescence
Source: PLoS Comput Biol. 2021 Nov 8;17(11):e1009584. doi: 10.1371/journal.pcbi.1009584 (PMC8601617; doi:10.1371/journal.pcbi.1009584)
Supplement: Attachment — Submitted filename: RefReport_LechonPawar_2021.pdf [file pcbi.1009584.s002.pdf]

Referee Report
The role of competition versus cooperation in microbial community coalescence, by Lechón, P. et al.

Alberto Pascual-García

July 6, 2021

Institute of Integrative Biology, ETH-Zürich.
Universitätstrasse 16, 8005. Zürich, Switzerland.
Email. alberto.pascual.garcia@gmail.com

In this article, Pablo Lechón and collaborators study with the help of a consumers-resources mathematical model the outcome of the encountering of microbial communities (community coalescence) under different parameterizations and network topologies. They propose a quantity they term community-level cohesion, Θ, which allow them to predict which community will dominate the encountering, by quantifying the difference in cohesion of the parent communities. They main finding is that more cohesive communities tend to dominate the encountering.

The question the authors address is important and has an increasing interest in the field, the approximation is original and the results are interesting and potentially impactful. There are, however, a number of questions precluding me to recommend its immediate publication. Although the results seem to clearly be pointing towards the importance of cohesion in community-level encountering, there are internal inconsistencies and potential discrepancies with the existing literature, regarding *the mechanisms* explaining their results. More specifically, how "The role of competition versus cooperation", as the title reads, is interpreted in their results. The origin of my concerns comes from i) a lack of information to understand some results in specific steps of their methods; ii) the definitions of "competition" and "cooperation" in their model; and iii) some technical questions such as some choices they made or additional analysis missing, that may influence the previous points.

Summary of Main Points

To discuss my main concerns I am going to do something in poor form because I will refer to some of my publications, simply because it helps me to develop the arguments (some definitions can be found there) and because I would like to be completely transparent about my own biases, so I will sign the Report. The authors should not feel obliged to cite any of them if they don't find them useful for their paper.

In LV systems, the objects encoding competition, mutualism, etc., are explicitly defined in the ODEs. This is an important advantage with respect to other models and possibly one of the reasons of its popularity, since changes in these objects have an immediate interpretation. Still, there is a long history of controversy discussing what is the relative role of ecological interactions in, e.g., the stability of ecosystems, and much of this discussion arises from the different modelling choices. Nowadays, the large body of theory around this model allow us to link very explicitly objects such as the effective competition, which can encode all types of interactions present in the system, with properties of the system such as its global stability [1].

In the model the authors are dealing with, however, such connections are not so clear, since competition is indirect through consumption of resources. The authors define $\mathcal{C}$ and $\mathcal{F}$ and they refer to these quantities as "competition" and "facilitation", respectively. Although there is no doubt that $\mathcal{C}$ describes the resources overlap and hence it is proportional to the competition, we should be sure that these definitions are correct, since

the ecological interpretation arises from them. In addition, whatever it is the definition selected, we should understand how these definitions are related to feasibility and dynamical stability, to rule out potential "side-effects" responsible for the observed behaviour. For instance, the authors state that "minimizing competition ensures coalescence success", and we should note that the minimization of competition does not only come from minimizing resource consumption overlap, but from the indirect (positive) effect of facilitation. This has been shown for mutualistic systems, for which mutualism increases structural stability by minimizing the effective competition [2]. However, in the second section of Results the title states "cooperation undermines coalescence success" which contradicts previous reasoning (since now facilitation has a negative effect), and they just refer to some empirical references without further mechanistic investigation. And there are many possible reasons explaining this result (I suggest some below) and a very economic one, which is that the definitions selected for competition and facilitation are inaccurate. Of course, it would also be possible that everything is right and that there are two different regimes in which facilitation acts differently on the stability of the system. We found such behaviour in Ref. [3], but we found that mutualism could be detrimental exactly in the opposite regime the authors describe —for high competition, while the authors describe very low competition.

Therefore, I think it is important that the authors provide an analysis on how the different choices of parameters influence feasibility and dynamical stability, and which is the connection with competition and facilitation, or at least that they provide evidence that they are sufficiently controlling them. For example, an important point regarding the parent communities is that the authors do not mention if they are verifying that the assembled communities are dynamically stable. It seems to me they are implying that by, integrating the system, feasibility implies dynamical stability. I am not aware of an analytical result for this system as it is for LV, so I would like to ask them to provide analytical or numerical verification. This is important, in particular for those communities with high $\mathcal{F}$ values, which may be trivially disrupted in the encountering simply because they are not dynamical stable despite being feasible. Feasibility itself is another potential confounding factor. For example, in the discussion of equation S2 the authors state that positivity is ensured if $R_j - \chi_0 > 0$ but I don't think this is true, since the term $(1 - l)$ does not multiply z_α, and hence we should expect that the feasible space shrinks for higher values of l. I would expect that, by having more facilitation, the system becomes less feasible (because there are more constraints to be fulfilled). Hence, if the feasible space is very small due to our parameter choices, when we combine both communities the combination will more likely be unfeasible if there is more facilitation. Is this due to facilitation? One may argue that, given the conceptual relatedness of l and χ_0, a fair comparison among systems with different l values should also consider different χ_0 values, in a way in which the feasible space is comparable. Otherwise, we may attribute to the interactions an effect which is rather related with our modelling choices (here, a constant χ_0).

Specific Points

I will follow the text to provide feedback on specific points. Some of them are related to the two main points listed above and others should be considered minor.

Abstract

Please give context explaining why "Encounters between microbial communities are becoming increasingly frequent across the globe"

Introduction

- Line 18. Since dominance is asymmetric by definition, I think more context should be given for those not familiar with the publication.
- Line 28. I and collaborators suggested this possibility in Ref 35 for communities termed "metabolically cohesive consortia", please consider referencing it here.
- Line 33. "efficient resource"... "consumption" may be missing?

- Line 33. "the invasion success of a given taxon is determined by its community members" how? please provide more context.

Methods

- I would like to ask the authors to provide a more elaborated description of step 2 and 3 of their methodology, and to include some results in the Main Text. It is very unbalanced the description of step 1 Vs step 2 and 3 and the are some unclear steps (such as how dynamical stability was verified). I think that the assembly of parent communities is interesting per se, and much needed to understand downstream analysis. I can imagine that the authors may consider this analysis distracting, but given that there is no space limitation and that these results are also novel, I encourage the authors to dedicate a specific section in Results. The first thing I think is unclear is if the quantities they present as describing the "parent communities", refer to the "starting communities" (before the assemblage) or to the assembled communities. More specifically, I would like to ask the authors to provide the summary statistics for Θ, $\mathcal{C}$, $\mathcal{F}$, species richness (and perhaps true diversity, i.e. the exponential of the Shannon diversity), and metrics describing the abundances of the communities at steady state for starting, assembled and final communities (e.g. mean and CV). Some of these metrics may be as predictive as Θ or they may have a relevant influence. Since cohesion depends on some of the metrics I am asking the authors to compute, and they have a more direct interpretation, we would like to discard they have a more relevant influence than cohesion. Some of the caveats I mentioned above regarding dynamical stability and feasibility of parent communities could also be addressed in this section.
- Equation 1. As I anticipated I haven't found the specific values the authors used for some parameters, in particular κ, g and χ_0. Please consider providing a table with all parameters (including k_c, K_c, etc), their values, meaning, and any other useful information.
  - Although the model was first proposed elsewhere, I would acknowledge if the authors justify their choice of the functional form for the increase in resources due to metabolic by-products. More specifically, why the term contains the resources abundances. For instance, if we consider metabolic by-products from central metabolism, the secretion would be proportional to species densities only, as suggested in Ref. [4].
  - I do not see why the authors find justified to remove the dilution term for resources, please justify.

Competition and facilitation metrics. I tried to follow the author's reasoning in the metrics they propose and I am afraid I am not able to understand some of the arguments. The first thing is why it is needed a split between abiotic and biotic resources. It looks like the authors are trying a transformation of the system into an effective system, but I do not see the need and I think it is complicating things quite considerably. Firstly, if there is a split between abiotic and biotic resources it means that some of the κ parameters are zero, a possibility that should be stated from the beginning. Then, a new parameter should be considered to quantify the fraction of resources that are externally supplied and understand its consequences for feasibility, dynamical stability, etc. I think that a microbe just "sees" a resource with some abundance, no matter its origin. Therefore, if species α consumes resources at rate $(1-l)c_{\alpha k}$ and species β at rate $(1-l)c_{\beta k}$, I would expect defining competition by the product:

$$C_{\alpha\beta} = (1-l)^2 \sum_k c_{\alpha k} c_{\beta k},$$

and similarly for facilitation

$$F_{\alpha\beta} = l(1-l) \sum_{jk} c_{\alpha j} D_{jk} c_{\beta k}.$$

This would be somewhat the "structural" competition and facilitation at each time-step of the simulation, and the simulation will tell us the effects, so I do not see the need, for instance, for considering the infinite loop of cycles (moreover, if it is considered for $l(1-l)$ it should also be considered for the object $c_{\alpha j} D_{jk} c_{\beta k}$).

Another thing I do not understand is the inclusion of $\tilde{\kappa}$ (possibly for the same reason I do not understand the need for the split of resources). But even if this distinction is needed, according to its definition, competition will be stronger for resources with high supply, and one could argue that species competing for only one available resource experience higher competition if the resource is scarce.

Then the authors average out the values of the matrix but they exclude diagonal values. However, I do not see why should be excluded since it is very likely an important component of the stability of the system (think about diagonal dominance in LV systems), even more if we aim to compare the relative role of competition and facilitation. This leads me to another important questions regarding the design and parameterization of the matrices. I think it should be clear whether intraspecific syntrophy is considered (i.e. that a species consumes what it secretes). Although I can imagine that there are certain situations in which this has been observed when conditions change (similar to diauxie growth), in this model the metabolic strategies are fixed independently of the environmental conditions so I do not see it justified to consider this possibility, at least in general.

The last point in this section I would ask the authors to reconsider is that, by averaging across all pairs of species, the definition represents somewhat a mean-field competition value. But I think this may not be justified for the guild structure, which is far from mean field. Since, between guilds, competition is zero, I think that the quantity should be computed for each guild independently and then averaged out across guilds. This I think would solve the (to my taste artificially low values) arising from many pairs with no competition. Moreover, since competition is harsh within guilds, I would expect that the outcome of the encountering would exclude the species feeling stronger competition within each guild (a prediction we present in Ref. 35, see Fig 4a which is the author's guild structure and Fig 4b and 5 for possible outcomes). This could be verified computing how the species extinctions are distributed across guilds.

Simulations

- Equation 7. Since α is used for both species and iterations it is confusing if it is not clarified (as the authors present in the SM).
- My understanding of the procedure is that the authors adapted the preferential attachment model of Barabási and Alberts to their problem, perhaps it is worth mentioning it in the beginning since it is a widely known model.
- The authors state that "the resources that are highly demanded are also secreted in large fractions". But if, for example, glucose is highly demanded, we will not expect glucose to be secreted in large fractions, but acetate. Might be they meant "the resources that are secreted in large fractions are highly demanded"? The question here is to be clear on whether a species is consuming what it secretes or not.

Results and Discussion

I think the results are clear and well-presented, and most of the things I would like to see in Results have already been discussed above. Some minor things.

- In Figure 3D I would perhaps find more interesting and easier to understand plotting in the y axis the species richness (and colour the dots by resource depletion level), since it would be more directly connected with the stability (x-axis) diversity (y-axis) problem (with the colour being the mechanism).
- Figure S2 top. How can the number of consumers of a resource increase after assembly? Typo in the caption "plots it the".
- SM Page 11. Typo "this communities".
- SM8. Please review this section, I think the first equation is not correct, if I am not wrong it should be $(\mathrm{diag}(g \circ n)\mathrm{CR})$. Also, l is a constant so no need for vector notation. Consider presenting the element-wise product $\circ$.

References

[1] Ferrera A, Pascual-García A, Bastolla U. Effective competition determines the global stability of model ecosystems. Theoretical Ecology. 2017;10(2):195–205.

[2] Bastolla U, Fortuna MA, Pascual-García A, Ferrera A, Luque B, Bascompte J. The architecture of mutualistic networks minimizes competition and increases biodiversity. Nature. 2009;458(7241):1018–1020. doi:10.1038/nature07950.

[3] Pascual-García A, Bastolla U. Mutualism supports biodiversity when the direct competition is weak. Nature Communications. 2017;8:14326.

[4] Butler S, OĎwyer JP. Stability criteria for complex microbial communities. Nature Communications. 2018;9(1):2970.
