# Supplementary material for: The role of competition versus cooperation in microbial community coalescence
Source: PLoS Comput Biol. 2021 Nov 8;17(11):e1009584. doi: 10.1371/journal.pcbi.1009584 (PMC8601617; doi:10.1371/journal.pcbi.1009584)
Supplement: Attachment — Submitted filename: response_to_reviewers.pdf [file pcbi.1009584.s003.pdf]

Responses to the Reviewers

Summary of Revisions

We thank all three reviewers for their detailed review and constructive comments, which have prompted us to make revisions that have greatly strengthened our study. Overall, five main issues were raised by the reviewers, which we have addressed as follows:

(i) *The parameter sensitivity of our results*: To address this, we have relaxed certain key assumptions and re-run the simulations, and added more detailed justifications for other parameter choices (especially of the binary uptake rates / consumer preferences)

(ii) *The need to better quantify properties of parent communities*: To address this, we have performed extensive new simulations and added a new section and figure that shows the key properties of parent communities (providing a better basis for relating them to coalescence outcomes)

(iii) *The effect of repeated coalescence events on community-level properties*: To address this, we added a new serial coalescence simulation procedure and correspondingly, a new results figure

(iv) *The validity of our community-level competition and cooperation measures and the need for more analytical insight into how they link to coalescence outcomes*: To address this, we have revised the competition and cooperation measures, which prompted us to add another type of coalescence procedure—recursive coalescence—to better address the role of cooperation in coalescence

(v) *The need for further analysis of the feasibility and stability of the parent and coalesced communities*: To address this, we have made key revisions to the model structure (that have rendered it more general), added a new analytical section to the SM, and extensive new supporting simulations

Our detailed, point-wise responses follow. Reviewers' original comments are in **black**, and our responses in **blue**. The revised manuscript with key changes highlighted, as well as an unmarked version, are attached.

Reviewer #1

1. Community cohesion is defined as the difference between facilitation and competition within a community. However, these metrics are computed on the initial pools, i.e. before the community assembly and stabilization steps. Because both competition and facilitation interactions can change during community assembly (e.g. species become extinct, modify their density etc) I am left wondering how the reported results would change if cohesion was measured in the stabilized communities.

   All properties were measured for initial (before assembly), parents (after assembly), and coalesced communities. We apologize for not being sufficiently clear previously, and have now revised the main text (Figure 1 text legend, and details of "Step 2: Assembly of parent communities", lines 155–157) to clarify this issue.

2. Similarly, the authors consider equal growth rates for all resources and species (e.g. binary C matrices). I am similarly left wondering how would relaxing this assumption and considering different growth rates for different species and resources impact the results.

   The model used in this paper is an extension of the one originally developed in Marsland et al. (2019). In the original work the authors considered the impact of two separate scenarios where deviations from equal growth rates would be observed. Firstly, the case

where the C matrices were either gamma or Gaussian distributed rather than being binary. Secondly, when the 'value' (w_i) of metabolites was allowed to vary rather than being set to one for all metabolites. More biomass growth occurs per amount uptaken for metabolites with a higher 'value'. In our model the value is implicitly set to one for all metabolites. The authors of the original paper found (see their Figs S1–6) that neither scenario significantly changed the ecosystem properties at steady state. As such we do not expect that relaxing this assumption would change the dynamics we observe significantly at the ecosystem level, though it would clearly have impacts at the species level. Our metrics (Eqs 5&7) are set up in such a way that they could account for non-binary C matrices, and would merely to be weighted by metabolite 'values' to account for variations in those. Therefore, we expect that our results relating coalescence success to the strength of competition and facilitation to be broadly unchanged if we consider scenarios more realistic than the equal growth scenario. A comment referring the interested reader to this previous work has been added to the Discussion section (lines 350–354).

Reviewer #2

Major comment

3. The results strongly focus on the success of parental communities at surviving the coalescence process. However, I was missing a comparison between the properties of the parental communities and the coalesced community. The only results shown in that regard are to be found in supplement's Fig S2. Are coalesced communities more cohesive than both parental strains, or something in between? Do facilitation/competition levels increase or decrease during coalescence? I think that there is an important discussion missing that could shed light on community-level properties that might, or might not, be optimized during coalescence. If we iterate coalescence for a few rounds (the survival species of two coalesced communities are exposed to a new coalescence event), what should we expect? What is the speed at which iterative coalescence leads to a sort of convergence in community structure? I think that adding a figure addressing (at least some of) these questions in the main text could improve the significance of the work.

   We thank the reviewer for this comment in particular as it has prompted us to generate new results that we feel strengthens the paper substantially. Specifically, we added a new type of coalescence simulation (Serial) which provide deeper mechanistic insights, including into the the community-level evolution of structural and functional properties. Please see lines 177–183 of the Methods, lines 247–261 of the Results, and lines 319–343 of the Discussion sections. In short, we find that repeated coalescence decreases competition, increases cooperation, and enhances invasion resistance of communities.

Minor comments

4. The second paragraph in the introduction seems to rush directly into the importance of interactions in coalescence. Around line 14, I think that it could be worth to introduce other potential drivers of coalescence. Just as possible examples to address: resource availability, species growth rates, dispersal rates (communities in the process of coalescence might still receive some degree of immigration from the environment), seasonality (or temperature, day-night light cycles, ...).

   Following the reviewer's suggestion, we have now widened mention of the scope of factors that can play an important role in coalescence (lines 14–21).

5. Eq. 2: wouldn't it be better to add subindexes to the noise term epsilon? Otherwise it looks like a universal constant in the equation. In any case, the supplementary text S2

could be a bit more precise about the parameters of the gaussian distribution from which epsilon is sampled, since the text in S2 only says that is a ‘small fluctuation term’. How much small? How sensitive the system is to this noise?

We have added a subindex to epsilon, since it is species-dependent. We have added details on the distribution from which ϵ is sampled in the new Supplementary material Section S1. Additionally, our results are conditional on the fact that epsilon is small (and would remain qualitatively unchanged if in fact these fluctuations were set to zero), which we now clarify in Supplementary material Section S1.1 .

6. The meaning of kappa in Eq 5 should be briefly introduced in the main text, even if it's explained in more detail in the supplement.

   We have changed the measures of competition and facilitation to address the comments of reviewers 2 and 3, which eliminates the parameter κ (see our responses to comments #22 and 23). In brief, competition and facilitation are not dependent on the external environment, but rather only on the preferences of the bacteria.

7. Before Eq. S4: why is it that consuming resource j is relevant to assess competition on resource k? It seems to me that a third species could be consuming j and leak k, and then the two focal species compete just for consuming k.

   This comment no longer applies, since the measures have been changed (see our response to previous comment and comments #22 and 23).

8. I also have a hard time at understanding why competition for leaked resources and supplied resources ‘need to be calculated differently’ (page 4 in the supplement), meaning separately. I understand that it's convenient to compute the two terms separately. Do the authors mean ‘can be calculated separately’ instead of ‘needs to be…’?

   We have now substantially simplified the measures of competition and facilitation (see responses to previous two comments and comments #22 and 23), such that we no longer separately calculate competition for leaked resources and externally supplied resources.

9. Why is it that (1-l) affects the metric for abiotic competition? In principle, microbes compete to uptake a full unity of a given resource from the environment. Once this happens, microbial metabolism converts (1-l) into population density, and a fraction ‘l’ is leaked in the form of other resources. But I don't think that competition itself should be weighted by the unleaked fraction in this case.

   The reviewer raises a very good point. We have eliminated the factor $(1-l)$ in the competition calculation, and substantially simplified the measure (see responses to previous three comments and comments #22 and 23).

10. The amount of parameters needed to describe the model makes it a bit hard for the reader to remember the meaning of each of them in the first reading. To make the paper more rapidly understandable at first glimpse, the authors could consider adding legends or equations for each term appearing on the figures. For example a legend in Fig 1 could include ‘cohesion = facilitation − competition’, ‘D = metabolic matrix’. Same for the meaning of ‘kc’, ‘Kc’ and ‘r’ in Fig 2 and 3.

    In order not to clutter the figures too much, we have avoided this. Instead, we have now added a table of parameters with definitions to the main text's Methods section.

11. In figure 3D, R*/r is named ‘Resource depletion level’. Through this name, I would understand that a higher value on this quantity translates to a lower R*. That is, communities with higher resource depletion levels should more efficiently deplete the resources. Unless

I am missing something, this is not how it is interpreted and, to me, it is a little bit counterintuitive in the present form.

Agreed - we have changed 'resource depletion level' to 'Resource abundance at equilibrium' in our revised figures.

12. I think that fig 4A is not referenced in the main text.

We thank the reviewer for their careful reading. We have now made sure that all figures in the main text are referred to.

Reviewer #3

Main Points

13. In LV systems, the objects encoding competition, mutualism, etc., are explicitly defined in the ODEs. This is an important advantage with respect to other models and possibly one of the reasons of its popularity, since changes in these objects have an immediate interpretation. Still, there is a long history of controversy discussing what is the relative role of ecological interactions in, e.g., the stability of ecosystems, and much of this discussion arises from the different modelling choices. Nowadays, the large body of theory around this model allow us to link very explicitly objects such as the effective competition, which can encode all types of interactions present in the system, with properties of the system such as its global stability [1]. In the model the authors are dealing with, however, such connections are not so clear, since competition is indirect through consumption of resources.
In the model the authors are dealing with, however, such connections are not so clear, since competition is indirect through consumption of resources.

The Reviewer has raised a very interesting and important point. Yes, an analytical understanding of this (new) class of (microbial) consumer resource models lags behind that of LV type models. As the reviewer says, derivation of the effective interaction matrix is a key step towards an in-depth analysis of the link between the structure and dynamics (especially, feasibility and stability) of these systems. Recent work has indeed made an advance in that direction (Marsland et al., 2020). We think that an in-depth analysis of our model based on derivation of the effective interaction matrix is outside the scope of our paper. However, prompted by further helpful comments by the Reviewer, we have now performed an analysis of the model that addresses the link between interaction structure, feasibility, and coalescence outcomes, and provides deeper insights into our numerical results (next response). We also now check local stability of the assembled parent and coalesced communities (also explained in our response to the next comment).

14. The authors define $\mathcal{C}$ and $\mathcal{F}$ and they refer to these quantities as "competition" and "facilitation", respectively. We should be sure that the definitions of $\mathcal{C}$ and $\mathcal{F}$ are correct, since the ecological interpretation arises from them. In addition, we should understand how these definitions are related to feasibility and dynamical stability, to rule out potential "side effects" responsible for the observed behaviour. For instance, the authors state that "minimizing competition ensures coalescence success", and we should note that the minimization of competition does not only come from minimizing resource consumption overlap, but from the indirect (positive) effect of facilitation. This has been shown for mutualistic systems, for which mutualism increases structural stability by minimizing the effective competition Bastolla et al. (2009). However, in the second section of Results the title states "cooperation undermines coalescence success" which contradicts previous reasoning (since now facilitation has a negative effect), and they just refer to some empirical references without further mechanistic investigation. And there are many possible reasons

explaining this results (I suggest some below) and a very economic one, which is that the definitions selected for competition and facilitation are inaccurate. Of course, it would also be possible that everything is right and that there are two different regimes in which facilitation acts differently on the stability of the system. We found such behaviour in Pascual-García and Bastolla (2017), but we found that mutualism could be detrimental exactly on the opposite regime the authors describe – for high competition.
I think it is important that the authors provide an analysis on how the different choices of parameters influence feasibility and dynamical stability, and which is the connection with competition and facilitation, or at least that they provide evidence that they are sufficiently controlling them. For example, an important point regarding the parent communities is that the authors do not mention if they are verifying that the assembled communities are dynamically stable. It seems to me that they are implying that by, integrating the system, feasibility implies dynamical stability. I am not aware of an analytical result for this system as it is for LV, so I would like to ask them to provide analytical or numerical verification. This is important, in particular for those communities with high $\mathcal{F}$ values, which may be trivially disrupted in the encountering simply because they are not dynamically stable despite being feasible. Feasibility itself is another potential confounding factor. For example, in the discussion of equation S2 the authors state that positivity is ensured if $R_j - \chi_0 > 0$ but I don't think this is true, since the term $1 - l$ does not multiply z_α, and hence we should expect that the feasible space shrinks for higher values of l. I would expect that, by having more facilitation, the system becomes less feasible (because there are more constraints to be fulfilled). Hence, if the feasible space is very small due to our parameter choices, when we combine both communities the combination will more likely be unfeasble if there is more facilitation. Is this due to facilitation? One may argue that, given the conceptual relatedness of l and χ_0, a fair comparison among systems with different l values should also consider different χ_0 values, in a way in which the feasible space is comparable. Otherwise, we may attribute to the interactions an effect which is rather related with our modelling choices (here, a constant χ_0)

The Reviewer has raised several important points here, which we address in turn:

- *Definitions of the competition and facilitation measures*: We have now revised the definitions of $\mathcal{C}$ and $\mathcal{F}$ - please see comment #22 below (and main text lines 101–117). This also means that we now have a new and simpler measure ($\mathcal{C} - \mathcal{F}$) which we call "net competition" that well-predicts coalescence outcomes.

- *The link between the interaction structure measures and coalescence*: The Reviewer's comment prompted us look more closely at the mathematical model and perform additional analyses. As the reviewer correctly noticed, we found that whether leakage is included in the consumer species' cost function matters to coalescence outcomes. Specifically, if the cost is independent of leakage level, communities with higher leakage are unable to deplete resources to a concentration as low as communities with lower leakage, ensuring that less leaky community would dominate in pairwise coalescence events. Therefore, we further generalized our model and analyses to consider the effect of varying the cost function (detailed in new SM section S1.2). Doing so shows that if leakage is included in the cost function (and which is also biologically realistic) it eliminates the inherent coalescence bias towards less leaky communities. This substantially clarifies the role of competition vs cooperation in community coalescence (see lines 265–273 in Discussion), and also help reconcile our results with the previous research cited by the Reviewer that cooperation enhances coexistence, but conditional on direct competition levels (Bastolla et al. (2009); Pascual-García and Bastolla (2017). We now also mention this in the Discussion (lines 305–307 & 286–289).

- *The issue of feasibility*: In a model community under the revised mathematical model (see response to previous comment), feasibility is now independent of leakage because of the new cost function (see main text lines 85–94, SM section S1.1, and previous answer). This allows us to fairly coalescence communities with different leakage and correctly determine the role of cooperative interactions in the outcome of community coalescence.
- *The issue of stability*: We have now checked that all parent as well as coalesced communities are locally stable (described in new SM section S1.3, and mentioned in lines 199–201 of the revised main text).

Specific Points

Abstract

15. Please give context explaining why "Encounters between microbial communities are becoming increasingly frequent across the globe"

    We have now added more rationale for this sentence (2nd from last sentence in the abstract).

Introduction

16. Line 18: Since dominance is asymmetric by definition, I think more context should be given for those not familiar with the publication.

    We have removed the word "asymmetrical", since the reviewer is right in that "dominance" is asymmetrical by definition. See new explanation in lines 22–24.

17. Line 28. I and collaborators suggested this possibility in Ref 35 for communities termed "metabolically cohesive consortia", please consider referencing it here.

    This reference was a significant influence when we were conceptualising the study performed in this manuscript. We have included an additional reference to it as suggested (lines 29–31).

18. Line 33. "efficient resource"... "consumption" may be missing?

    We definitely missed a word here. We have now added "use", as this is the terminology used in the reference (line 36).

19. Line 33: "the invasion of a given taxon is determined by its community members" how? please provide more context.

    We have now clarified this (lines 36–39)

Methods

20. I would like to ask the authors to provide a more elaborated description of step 2 and 3 of their methodology, and to include some results in the Main Text. It is very unbalanced the description of step 1 Vs step 2 and 3 and there are some unclear steps (such as how dynamical stability was verified). I think that the assembly of parent communities is interesting per se, and much needed to understand downstream analysis. I can imagine that the authors may consider this analysis distracting, but given that there is no space limitation and that these results are also novel, I encourage the authors to dedicate a specific section in Results. The first thing I think is unclear is if the quantities they present as describing the "parent communities", refer to the "starting communities" (before the

assemblage) or to the assembled communities. More specifically, I would like to ask the authors to provide the summary statistics for Θ, $\mathcal{C}$, and $\mathcal{F}$, species richness (and perhaps true diversity, i.e. the exponential of the shannon diversity), and metrics describing the abundances of the communities at steady state for starting, assembled and final communities (e.g. mean and CV (coefficient of variation, a measure of relative variance in abundance around the mean)). Some of these metrics may be as predictive as Θ or they may have a relevant influence. Since cohesion depends on some of the metrics I am asking the authors to compute, and they have a more direct interpretation, we would like to discard they have a more relevant influence than cohesion. Some of the caveats I mentioned above regarding dynamical stability and feasibility of parent communities could also be addressed in this section.

We agree, and have now added more explanation of the assembly of the parent communities and their structural and dynamical properties (see response to comment #14 above with regards to stability analyses, lines 199–201; and Methods, Step 2, lines 139–157). We have also added new results section describing key properties of the parent communities before and after their assembly (lines 203–228).

21. Equation 1. As I anticipated, I haven't found the specific values the authors used for some parameters, in particular κ, g, and χ_0. Please consider providing a table with all parameters (including k_c, K_c, etc), their values, meaning, and any othger useful information.

    We have added a table (Table 1) of parameters with definitions to the main text.

    (a) Although the model was first proposed elsewhere, I would acknowledge if the authors justify their choice of the functional form for the increase in resources due to metabolic by-products. More specifically, why the term contains the resources abundances. For instance, if we consider metabolic by-products from central metabolism, the secretion would be proportional to species densities only, as suggested in Butler and O'Dwyer (2018).

    We have now added justification in the main text in lines 74–83 for our choice of leakage dependency which rests on two points. Firstly we assume dependence of by-product formation in resource uptake as it reflects a much more general feature of microbial communities in nature. The leakage of by-products has been observed to be important in microbial communities Goldford et al. (2018) and in fact even the authors in the paper referred to by the reviewer note that their assumption of consumer mass dependence is only a "reasonable approximation for some processes". Secondly we assume dependence as it allows a much wider range of cross-feeding interactions between individual consumer pairs. Under the consumer-mass dependence assumption positive interactions are global such that each individual consumer benefits the same from the other consumers in the system. As the main aim of this paper is to establish the effects of interactions and their specific structure on community coalescence such an assumption is too restrictive and would not allow full range of effects of cross-feeding to be explored.

    (b) I do not see why the authors find justified to remove the dilution term for resources, please justify.

    We had originally dropped the dilution term to make the model more comparable to that of Tikhonov (2016). However, we agree with the reviewer that the choice is somewhat arbitrary even if it does emulate certain natural (e.g., in an isolated soil pocket) or experimental (e.g., single vial experiments without serial dilution) scenarios where resources are not diluted. Therefore we have now also generated results for chemostat type systems (with resource dilution), and show that the assembly and

coalescence results don't qualitatively change due to adding this parameter. The only difference is that the number of simulations that converge when there is a dilution term increases because resources that are not consumed by any species will not diverge (as they did in the absence of dilution), but reach a carrying capacity determined by the ratio between κ and τ. We now show only the results for this altered model in the paper.

Competition and facilitation metrics

22. Why it is needed a split between abiotic and biotic resources. It looks like the authors are trying a transformation of the system into an effective system, but I do not see the need and I think it is complicating things quite considerably. Firstly, If there is a split between abiotic and biotic resources it means that some of the κ parameters are zero, a possibility that should be stated from the beginning. Then a new parameter should be considered to quantify the fraction of resources that are externally supplied and understand its consequences for feasibility, dynamical stability, etc. I think that a microbe just "sees" a resource with some abundance, no matter its origin. Therefore, if species α consumes resources at rate $(1-l)c_{\alpha k}$ and species β at rate $(1-l)c_{\beta k}$, I would expect defining competition by the product:

$$C_{\alpha\beta} = (1-l)^2 \sum_k c_{\alpha k} c_{\beta k},$$

and similarly for facilitation

$$F_{\alpha\beta} = l(1-l) \sum_{jk} c_{\alpha j} D_{jk} c_{\beta k}.$$

This would be somewhat the "structural— competition and facilitation at each time-step of the simulation, and the simulation will tell us the effects, so I do not see the need, for instance, for considering the infinite loop of cycles (moreover, if its considered for $l(1-l)$ it should also be considered for the object $c_{\alpha j} D_{jk} c_{\beta k}$.

The reviewer raises very good points. The separation between abiotic and biotic resources is unnecessary, and only serves to make things more complicated. Therefore have now simplified our competition and facilitation metrics, making them more parsimonious and intuitive (lines 101–117). Additionally, some of the changes made to the metrics also follow the recommendations of a related point raised by Reviewer 2 (see above comments # 7–9 and our response).

23. I do not understand the inclusion of $\tilde{\kappa}$ (possibly for the same reason I do not understand the need for the split of resources). But even if this distinction is needed, according to its definition, competition will be stronger for resources with high supply, and one could argue that species competing for only one available resource experience higher competition if the resource is scarce.

The reviewer is right; including $\tilde{\kappa}$ was an attempt to transform the system into an effective system. However, it is more rigorous to state simple measures of competition and facilitation, and let the simulations tell us the effect. Consequently, $\tilde{\kappa}$ has been removed from our metrics.

24. The authors average out the values of the matrix but they exclude the diagonal values. However, I do not see why should be excluded since it is very likely an important component of the stability of the system (think about diagonal dominance in LV systems), even more if we aim to compare the relative role of competition and facilitation.

We agree that this is an important issue to clarify. Therefore, we have changed our definitions of $\mathcal{C}$ and $\mathcal{F}$ to take into account intraspecific competition and facilitation. We now include these terms when calculating community-level competition and facilitation, finding that this they do not qualitatively change the results (see Fig 3C). We also agree that understanding the contribution of these intraspecific interaction values to the dynamics of these systems is important, but with reference to our comment # 13 above, that would require us to calculate the effective interaction matrix, which we think is an avenue for future work.

25. It should be clear whether intraspecific syntropy is considered (i.e. that a species consumes what it secretes). Although I can imagine that there are certain situations in which this has been observed when conditions change (similar to diauxic growth), in this model the metabolic strategies are fixed independently of the environmental conditions so I do not see it justified to consider this possibility, at least in general.

    Consumption of leaked resources is a function present in the original consumer resource model upon which ours is based (Marsland et al., 2019). Leakage of essential metabolic resources back into the environment is a pervasive phenomenon in microbes (Morris, 2015; Kallus et al., 2017). Whilst it may seem counter-intuitive for microbes to secrete metabolites that they also consume, it has been suggested that such leakage can provide short-term growth benefits to bacteria, as a flux control or growth-dilution mechanism (Yamagishi et al., 2020*a*,*b*). We had previously mentioned this briefly in the discussion, but have now expanded further on this point, both in the Discussion (lines 295–303), and in the Methods where leakage in the model is described (lines 83–84).

26. By averaging across all pairs of species, the definition represents somewhat a mean-field competition value. But I think this may not be justified for the guild structure, which is far from mean field. Since, between guilds, competition is zero, I think that the quantity should be computed for each guild independently and then averaged out across guilds. This I think would solve the (to my taste artificially low values) arising from many pairs with no competition. Moreover, since competition is harsh within guilds, I would expect that the outcome of the encountering would exclude the species feeling stronger competition within each guild (a prediction we present in Ref 35, see Fig 4a which is the author's guild structure and Fig 4b and 5 for possible outcomes). This could be verified computing how the species extinctions are distributed across guilds.

    We have now calculated community level competition as the average over the block diagonals, and community level facilitation as the average over the off-block diagonals. In SM Section S4.1, figure S6, we show that the results are qualitatively robust to this modification.

Simulations

27. Equation 7. Since α is used for both species and iterations it is confusing if it is not clarified (as the authors present in the SM).

    We have changed the explanation of the simulation procedures, which can be found now in the Supplementary material S2.1.

28. My understanding of the procedure is that the author adapted the preferential attachment model of Barabási and Albert to their problem, perhaps it is worth mentioning it in the beginning since it is a widely known model.

    We were not thinking of the preferential attachment model when we developed this procedure. However, we realize now the clear similarity and therefore have added a statement

noting the similarity and a reference to Barabási and Albert's original paper (see supplementary text section S2.1).

29. The authors state that "the resources that are highly demanded are also secreted in large fractions". But if, for example, glucose is highly demanded, we will not expect glucose to be secreted in large fractions, but acetate. Might be they meant "the resources that are secreted in large fractions are highly demanded"? The question here is to be clear on whether a species is consuming what it secretes or not.

    Our model is specified such that in highly facilitative communities ($k_f \to 1$), resources that are highly demanded by the community are also secreted in large fractions. We have now clarified in the text that this pertains to facilitative communities only (Supplementary text S2.1) . The metabolic matrix D specifies the by-product leakage as a proportion of resource j which is transformed into resource k. It is therefore possible for species in our model to consume what they secrete, if their consumer preference matrix encodes for the by-product of another resource that they utilise. Ultimately the relative simplicity of our model, which does not contain a true stoichiometric matrix, prevents us from analyzing our results in terms of real metabolites.

Results and Discussion

30. In Figure 3D I would perhaps find more interesting and easier to understand plotting in the y axis the species richness (and colour the dots by resource depletion level), since it would be more directly connected with the stability (x-axis) diversity (y-axis) problem (with the colour being the mechanism)

    We agree; this has been changed in the revised manuscript (see main text Figure 3).

31. Figure S2 top. How can the number of consumers of a resource increase after assembly? Typo in the caption "plots it the"

    Corrected. Additionally, figure S2 has been removed, since we have included a section (lines 203–228) on community assembly in the main text that addresses the similar issues that Fig S2 was addressing.

32. SM Page 11. Type "this communities"

    Corrected.

33. SM8. Please review this section, I think the first equation is not correct, if I am not wrong it should be (diag($g \circ n$)CR). Also, l is a constant so no need for vector notation. Consider presenting the element-wise product $\circ$

    The Reviewer is correct, and we changed the first line of the equation (2nd set of equations in SM section S1) to correct the error.

References

Bastolla, U., Fortuna, M. A., Pascual-García, A., Ferrera, A., Luque, B. and Bascompte, J. (2009), 'The architecture of mutualistic networks minimizes competition and increases biodiversity', *Nature* **458**(7241), 1018–1020.

Butler, S. and O'Dwyer, J. P. (2018), 'Stability criteria for complex microbial communities', *Nature Communications* **9**(1).
URL: *http://dx.doi.org/10.1038/s41467-018-05308-z*

Goldford, J. E., Lu, N., Bajić, D., Estrela, S., Tikhonov, M., Sanchez-Gorostiaga, A., Segrè, D., Mehta, P. and Sanchez, A. (2018), 'Emergent simplicity in microbial community assembly', *Science* **361**(6401), 469–474.

Kallus, Y., Miller, J. H. and Libby, E. (2017), 'Paradoxes in leaky microbial trade', *Nature Communications* **8**(1).

Marsland, R., Cui, W., Goldford, J., Sanchez, A., Korolev, K. and Mehta, P. (2019), 'Available energy fluxes drive a transition in the diversity, stability, and functional structure of microbial communities', *PLoS Computational Biology* **15**(2), e1006793. doi: 10.1371/journal.pcbi.1006793.

Marsland, R., Cui, W. and Mehta, P. (2020), 'The minimum environmental perturbation principle: A new perspective on niche theory', *American Naturalist* **196**(3), 291–305.

Morris, J. J. (2015), 'Black Queen evolution: The role of leakiness in structuring microbial communities', *Trends in Genetics* **31**(8), 475–482.
URL: *http://dx.doi.org/10.1016/j.tig.2015.05.004*

Pascual-García, A. and Bastolla, U. (2017), 'Mutualism supports biodiversity when the direct competition is weak', *Nature Communications* **8**.

Tikhonov, M. (2016), 'Community-level cohesion without cooperation', *eLife* **5**, e15747. doi: 10.7554/eLife.15747.

Yamagishi, J. F., Saito, N. and Kaneko, K. (2020*a*), 'Advantage of Leakage of Essential Metabolites for Cells', *Physical Review Letters* **124**, 048101.
URL: *https://doi.org/10.1103/PhysRevLett.124.048101*

Yamagishi, J. F., Saito, N. and Kaneko, K. (2020*b*), 'Microbial Potlatch: Cell-level adaptation of leakiness of metabolites leads to resilient symbiosis among diverse cells', *bioRxiv* pp. 1–17.
