# Supplementary material for: The role of competition versus cooperation in microbial community coalescence
Source: PLoS Comput Biol. 2021 Nov 8;17(11):e1009584. doi: 10.1371/journal.pcbi.1009584 (PMC8601617; doi:10.1371/journal.pcbi.1009584)
Supplement: Attachment — Submitted filename: responses_to_reviewers.pdf [file pcbi.1009584.s004.pdf]

Responses to the Reviewers

We thank all reviewers for their kind comments. Our responses to reviewer 3's concerns follow. Original reviewer's comments are in **black**, and our responses in **blue**. The revised manuscript with key changes highlighted, as well as an unmarked version, are attached.

Reviewer #3

1. Lines 76-83. I do not find convincing the authors' arguments convincing regarding their choice of the functional form for the secretion of metabolites. In Dwyer's model, it would be perfectly possible to select appropriate consumption and secretion matrices to account for points i) and ii). For me, the difference is whether secretion of resources is proportional to the growth rate of the species or to their abundances. Since this point is beyond the scope of the article, I would suggest removing this explanation.

   This explanation has been removed from the main text.

2. Lines 133-135 A brief explanation of the different factors (kc, kf, Kc, Kf) in the Main Text is needed, in particular providing an insight into how they relate to competition / cooperation.

   An explanation of the factors k_c and k_f, and its implications on competition and cooperation has been added to Step 1: Parametrization (lines 122–139). However, we have left the explanation of the K_c and K_f factors in the Supplement, since all the results regarding the structured scenario are in the supplementary text.

3. Table 1. Please include in the Table the units of N and R. Also, a comment on the transformation from moles of resource to energy/biomass is needed.

   Since Table 1 contains information about the parameters of the model, and N and R are not parameters, but state variables, we state their units when we present them in the main text (lines 54–56). We have addressed the transformation of moles of resource to energy and then biomass in the presentation of the model (lines 54–63). This has led us to identify two mistakes in the units of the conversion factor g_α, and energy uptake rates $c_{\alpha j}$ which have now been amended (Table 1). We thank the reviewer for this.

4. Lines 148-149 I find it difficult to interpret this quantity and the abundances proportion. Firstly, it is not completely clear if are computed considering all the simulations aggregated or if it was computed for each simulation and then averaged (which, with the s.e. I think it would perhaps be more informative). The second thing is that we don't know which is the distribution of nr, which I think it is needed to understand Fig 2B. For example, it is not clear if it is the case that generalist species are more robust as stated in Results, or if it is just that (only) the more specialized species go extinct and that each species that has more nr preferences jumps to another (smallest) nr group. The most direct representation I can think of could be a heatmap with nr0 rows and nrFinal in columns (including nr = 0 as extinctions), and the color being the probability that a species in group nr0 ends up in group nrFinal .

   Firstly, we would like to clarify that the quantity is computed for each simulation and then averaged; this is now clarified in the caption of Figure 2 . Secondly, the distribution of m_r is exponential, with rate β. This is mentioned in the supplementary text, subsection S2.1. We have now clarified this by specifying the value of the rate of the exponential distribution. Based on the above comment (particularly "each species that has more nr preferences jumps to another (smallest) nr group"), we think the reviewer has misunderstood the meaning of m_r (due to our previously unclear explanation). The number of

metabolic preferences of a particular species does not change (we treat it as one of the features that define the species). What changes is the number of species with m_r metabolic preferences (due to extinctions during assembly). We apologize for this confusion, and now explain this more clearly in the main text (lines 157–160). With this in mind, we believe that the current representation appropriately captures the information we want to convey.

5. Fig 2 Caption: "communities are significantly more cooperative" I would say that this appears to be true for kc = 0.9 (although difficult to say for kf = 0.99), did the authors perform a statistical test to state that it is significant? Moreover, it is not possible to tell anything just from this figure for kc = 0.01.

   We did not perform statistical tests to state that it is significant. Therefore, we have now reworded the caption in Figure 2.

6. Fig 2. Please provide the formula used to compute the Inset (perhaps as SM).

   We have included an equation specifying how to calculate the weights used to plot figure 2B (inset) in Assembly of parent communities in the main text (Eq 5), and refer to it in "Assembly of parent communities" section of the Results (lines 225–227)

7. Caption Fig. S3. Please indicate the order of magnitude of the leading eigenvalue, since the scale covers several orders of magnitude it would be said that it is zero otherwise.

   We have included the values of the leading eigenvalues in both examples shown in the SM. See Figure S3 caption.

8. I appreciate the efforts made in the SM to explain with pseudocode the algorithms, but please push the new code in the repo. Also, I would like to suggest creating a release and permanently storing it in Zenodo for making it citable, see: `https://guides.github.com/activities/citable-code/`

   We apologize for this oversight. The updated code has now been pushed to the repository. We will provide a citable Zenodo release of the code upon manuscript acceptance.
